# The knowledge, barriers and opportunities to improve nutrition and physical activity amongst young people attending an Australian youth mental health service: a mixed-methods study

**DOI:** 10.1186/s12913-022-08182-0

**Published:** 2022-06-17

**Authors:** Tamieka Mawer, Katherine Kent, Andrew D. Williams, Courtney J. McGowan, Sandra Murray, Marie-Louise Bird, Sibella Hardcastle, Heather Bridgman

**Affiliations:** 1 Centre for Rural Health, University of Tasmania, Locked Bag 1322, Launceston, Tasmania 7250 Australia; 2 School of Health Sciences, University of Tasmania, Launceston, Tasmania Australia; 3 School of Health Sciences, Western Sydney University, Kingswood, New South Wales Australia; 4 Sport Performance Optimization Research Team, School of Health Sciences, College of Health and Medicine, University of Tasmania, Launceston, Tasmania Australia

**Keywords:** Mental health, Service delivery, Youth, Adolescents, Regional programs, Community nutrition programs, Community exercise programs, Community physical activity programs

## Abstract

**Background:**

Mental illnesses are the leading cause of disability in young people, and lifestyle interventions in young people at risk of mental illness remain a priority. Opportunities to improve nutrition and physical activity among young people through youth mental health services remain unclear. This study aimed to determine the knowledge and behaviors towards nutrition and physical activity, the barriers and enablers to improving behaviors, and the preferred providers and sources of information for nutrition and physical activity among a sample of young people attending a youth mental health service.

**Methods:**

A mixed-method study was conducted in regional Tasmania, Australia in a sample of young people (15–25 years) attending a youth mental health service (*headspace*). A quantitative survey (*n* = 48) determined young people’s nutrition and physical activity knowledge, behaviors, barriers and enablers to achieving recommendations, and their preferred providers and sources of information. Structured interviews and a focus group further explored these concepts (*n* = 8), including the role of the mental health service as a provider of this support.

**Results:**

The majority of participants did not meet national recommendations for nutrition and physical activity, despite possessing a high level of knowledge regarding their importance for mental health. Improving mental health was a common enabling factor for participants choosing to alter diet and physical activity habits, but also the leading barrier for participating in physical activity. Young people wanted to receive information from reputable health providers, ideally through social media sources. *headspace* was seen as an important potential provider of this information.

**Conclusions:**

Our results indicate that there is a clear need to improve diet and physical activity habits to enhance mental and physical health outcomes in this at-risk group, and youth mental health services could provide further interventions to support their clients. Specialized staff (e.g. dietitians and exercise physiologists) may provide additional benefits alongside existing mental health care support.

**Supplementary Information:**

The online version contains supplementary material available at 10.1186/s12913-022-08182-0.

## Background

Young people (12–24 years) make up 13% of Australia’s population [1]. This is a key developmental period and a critical time for establishing good physical and mental health habits. When young people are in good health, they can more successfully transition into full-time work, develop healthy lifestyles, and experience fewer negative challenges in life [2]. The global onset of a first mental disorder occurs before age 14 in 34.6% of individuals, increasing to almost half (48.4%) by age 18 and extends to 62.5% by age 25, indicating that most adult mental disorders originate earlier in the lifespan [3]. Worldwide, mental illnesses cause more disability than any other illness in young people [4].

Good nutrition is essential for supporting mental health [5]. However, young people have the lowest diet quality compared with any other age group [6]. Poor dietary habits are more prevalent in individuals with mental health issues. Poorer food choices [7], medications [8], lifestyle behaviors [9], lower social determinants and behavioral problems [10] are contributing factors. Unhealthy dietary patterns have been associated with worse mental health in young people [11]. It has been theorized that young people may either eat poorly as a form of self-medication, or that poor quality diets lack sufficient nutrient-dense foods, leading to nutrient deficiencies associated with mental health issues [11] or affecting the development of the brain [12, 13]. Conversely, studies also suggest that a good quality, healthy diet, high in fruits and vegetables, can be protective of mental health [14, 15].

Further to the role of diet, regular participation in physical activity has been shown to improve mental health via changes to the structural and neurobiological composition of the brain [16]. Such changes encompass psychosocial mechanisms, including improved social connectedness, autonomy, self-acceptance, and mastery [17], and behavioral mechanisms, including improved coping skills and sleep hygiene [18]. There is growing evidence that physical activity is an effective treatment for people with acute and chronic mental illness, with evidence indicating that physical activity is just as effective, if not more effective than medications, in reducing depressive symptoms [19] as well as anxiety and stress symptoms [20]. Regular physical activity is recommended as a key element of mental health treatment [21]. Physical activity can improve mood and offset the negative side effects of common medications for mental illness, including improving body composition (lean muscle and reduced fat mass), blood pressure, cognition, and memory [22].

Knowledge and barriers to nutrition and physical activity in young people with mental illness:

Lower levels of knowledge regarding nutrition and physical activity recommendations have been associated with poorer dietary and physical activity behaviors in adults with mental illness [23]. However, the extent to which poor nutrition and physical activity knowledge contributes to poor dietary intake and lower physical activity levels in young people remains unclear. One small study of Australian young people with a mental illness found that nutrition knowledge scores were similar to those of other general population groups [24], suggesting that nutrition knowledge alone may not fully account for poorer dietary intake [24]. Thus, additional research is required to characterize the current knowledge of nutrition and physical activity in young people with mental illness to identify future opportunities to improve knowledge levels within this group.

The barriers and motivating factors towards achieving nutrition and physical activity recommendations in young Australian populations has been characterized [25]. Common motivators include improving health, body image and increasing energy, and common barriers relate to poor access to healthy food and exercise equipment, a real or perceived lack of time, tiredness/stress and a lack of motivation [26–29]. While some unique barriers and enabling factors have been identified in some different at-risk groups [30], the extent to which unique barriers and enablers affect young people with mental illness’s ability to eat healthily and participate in regular physical activity presently remains unclear.

Young people prefer to receive health information through a variety of sources, including their families, friends, the media and school [31]. It has been reported that increasingly, young people are turning to social media for information and support related to physical activity and nutrition habits [32]. However, the credibility of these sources is a challenge, and in particular, some young people have difficulty identifying which resources contain valid and reliable information [32]. The internet and mental health services are important sources of mental health support for people with mental illnesses [33]. However, the preferred sources of support and information for youth attending mental health services pertaining to nutrition and physical activity in this particular at-risk group remains unclear.

A growing body of evidence suggests that lifestyle behaviors, including nutrition and physical activity, can be substantially improved in those who attend mental health services using health promotion programs [34–36]. However, there remains limited research indicating how these principles can be applied to positively influence youth mental illness outcomes, which is vital given the higher prevalence of mental disorders in young people [3]. To improve the physical and mental health of young Australians with mental illness, effective programs focused on modifying nutrition and physical activity behaviors are urgently needed. Further, little research on the knowledge and attitudes towards nutrition and physical activity practices among young people attending a regional Australian youth mental health service has been published. Whilst there may be servicing challenges such as a lack of funding to run programs or to employ accredited nutrition and exercise practitioners to support clients, youth mental health services still require a clear understanding of the attitudes of young people towards the service’s role in providing activities which support healthy nutrition and physical activity practices. Without this, there is a risk that any implemented strategies will not meet the needs of their clients nor facilitate an improvement in their mental illness outcomes and management. Therefore, to inform future service delivery, the perspectives of young people attending a regional youth mental health service were investigated with the following research questions:

1. What are the behaviors and level of knowledge towards nutrition and physical activity in young people attending a regional youth mental health service?
2. What are the main barriers and enablers to achieving recommendations for nutrition and physical activity in young people attending a regional youth mental health service?
3. What do young people attending a regional youth mental health service understand about the relationship between nutrition and/or physical activity, and mental health outcomes?
4. What are the preferred providers and sources of information and support for nutrition and physical activity in young people attending a regional youth mental health service?

## Methods

### Study setting

Cornerstone Youth Services is based in Launceston, Tasmania, a regional city with a population of approximately 110,000 people [37]. In 2016, people aged 10–24 years comprised about 18% of the Tasmanian population [38]. Cornerstone delivers *headspace,* an Australia-wide non-profit organization that provides early intervention mental health services to 12–25 year olds. The service is designed to make it as easy as possible for a young person to access the help they need for problems affecting their wellbeing. *headspace* Launceston comprises a multidisciplinary team of clinicians, youth workers and administration staff. Young people can access services for free. The service has a *headspace* Advisory and Reference Team (hART) comprising young people that have previously accessed *headspace.* The hART is regularly consulted and offer youth perspective to inform service delivery and community engagement.

This study was a component of a larger project situated at *headspace* Launceston. The aim of the larger project was to increase overall service capacity to better support *headspace* clients to improve their nutrition and physical activity behaviors.

### Study design

A mixed methods study using a convenience sample was designed in consultation with *headspace* management, staff, and hART members to enable an in-depth gathering of the perspectives of young people regarding the role of nutrition and physical activity behaviors in the maintenance of good mental health. The study included a quantitative online survey, as well as structured one-to-one interviews and a focus group. A mixed methods study design was chosen as these studies offer in-depth qualitative understanding, providing rich data from which to develop patterns, themes and experiences [39] with the reach of quantitative measures [40]. This project has ethical approval by the Tasmania Social Science Human Research Ethics Committee (H0023475) and the study was conducted in accordance with the Declaration of Helsinki.

### Participants

Participants were young people aged between 15 and 25 years who self-identified as having had contact with *headspace* Launceston within the past 12 months or were members of the hART. Participants who completed the survey were given the opportunity to participate in a structured one-to-one interview. Members of the hART were also invited to participate in a structured focus group or one-to-one interviews.

### Recruitment

In consultation with the service, two recruitment methods were employed. First, *headspace* clinicians handed out flyers promoting the study to clients during their therapy sessions. Flyers were also available at reception. The flyers contained a study website link and a unique QR code enabling users to access the quantitative survey. Second, a five-week paid advertisement of the study was run on social media platforms to targeted youth in the area. Parameters on Facebook and Instagram were set to target 15–25 year-olds living in Launceston, Tasmania and surrounds. This resulted in 26,253 young people reached during the advertisement period. Data collection was undertaken between February and June 2021.

### Consent

As some participants were under the age of 18 years, an informed assent/consent process was designed. For the online survey all participants had to answer three screening questions to enter the survey to check their eligibility and understanding of the use of study data. For the interviews and focus group, consent was acquired in person or discussed over the phone with the interviewer (a clinical and health psychologist) to ensure the participants had appropriate cognitive capacity. All participants of the focus group and interviews signed a consent form.

### Online survey

The online survey (full survey supplementary file 1; summary of items in Table 1) was developed using adapted measures from existing validated tools, published surveys and guidelines specific to the study aims. *headspace* management and staff, hART members, and a high school teacher reviewed the survey for face validity and legibility. Based on the feedback, the survey was shortened to 33 questions and set at a reading level of age 12 to suit the known literacy level in the area, using Grammarly (2021© Grammarly Inc).

**Table 1:** Overview of the survey questions in the online survey for young people attending *headspace* in Launceston

Domain	Description of survey question	Literature source supporting the question
Nutrition	The frequency of consumption of standard serving sizes of fruit, vegetable, breakfast, water, sugary drinks and take-away foods.	Nutrition questions were based on recommendations for measuring nutrition and food consumption in children in Australia [41].
	Understanding of whether their frequency of consumption met Australian Guidelines regarding Healthy Eating recommendations for each food item.	Dietary intake questions were interpreted against the Australian Guidelines regarding Healthy Eating recommendations [42].
	Two nutrition knowledge questions about healthy eating and maintaining a healthy weight.	Adapted from a validated tool “General Nutrition Knowledge Questionnaire” [42].
Physical activity	Understanding of physical activity requirements for the maintenance of health and their current levels of physical activity in the context of the Australian government’s physical activity recommendations.	Australian government’s physical activity recommendations [43].
	Questions assessing the frequency and duration of physical activity.	The International Physical Activity Questionnaire [44], compared with the Australian government’s physical activity recommendations with results for those aged 15–17 years were compared against the children’s physical activity guidelines and those 18+ against the adult physical activity guidelines [43].
The role of nutrition and physical activity for mental health	Participants perceived importance of nutrition and physical activity behaviors for maintaining mental health.	Scale from 1 (not important) to 10 (important).
Accessing supporting	How participants currently access information and support about nutrition and physical activity and, how they would prefer to access this information in the future.	Tick box of pre-defined list of sources of support and information, developed from a literature search.
Barriers/Enablers	Barriers and enablers participants experience towards maintaining healthy eating and physical activity habits [45].	Tick box of pre-defined list of barriers and enablers, developed from a literature search.
Demographics	Age, gender, mental health diagnosis, *headspace* service access, and living situation.	

### Interviews and focus group

The interview and focus group guide was developed from literature review findings and questions related to the study aims developed by the research team to extend upon the concepts explored by the survey (See focus group schedule in supplementary file 2). Interviews and the focus group were held at the *headspace* office, by phone or online (e.g., via Zoom) at the preference of the participant and took 20–45 minutes. A clinical and health psychologist from the research team (HB) conducted all interviews. Interviews were transcribed into Microsoft Word and sent to the participants to review and make any changes if they wanted, within a two-week period. Once returned, the transcripts were de-identified for analysis.

### Data analysis

**Fig. 1:**
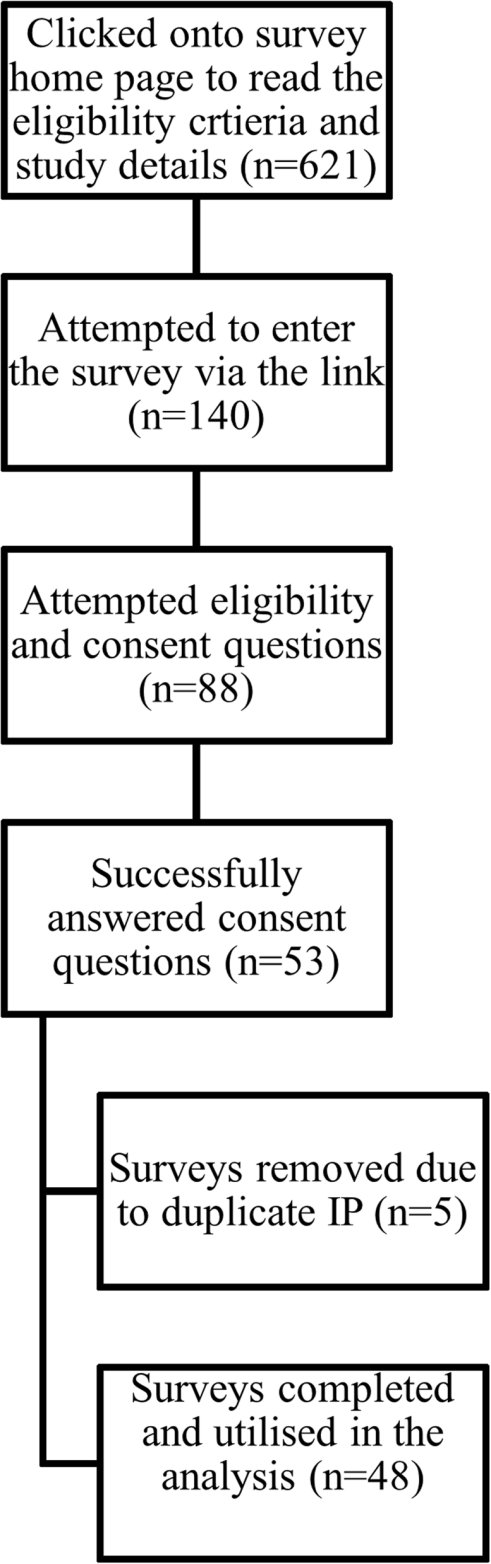
Overview of screening and consent process for the online survey

Online surveys were conducted through Lime Survey (limesurvey.org). An overview of the screening and consent process for the online survey is presented in Fig. 1. In total 621 potential participants clicked onto the survey home page, and 140 individuals clicked the survey link which led to the study information sheet and consent questions. Of those, 88 attempted the eligibility and consent questions which needed to be successfully answered to proceed to the survey and of these, 53 completed the survey. The remaining 35 were unable to provide consent. Duplicate surveys (surveys with the same IP address; *n* = 5) were removed prior to analysis. All data were downloaded and exported using Microsoft Excel (Microsoft Office Package 2011). All available data were analyzed using descriptive statistics (mean ± SD) in Microsoft Excel and presented in tables and figures.

Qualitative data was analyzed using an “inductive” approach [44], by two research team members (HB and AW). A coding guide was generated after familiarization with the data by the first researcher, and then codes were assigned to meaningful segments of text and agreed on in discussion between the two primary analysts. Codes were then grouped into categories, then sub-themes, and overarching themes were identified by discussion between the two researchers and then by team consensus.

## Results

Survey participants (*n* = 48) had a mean age of 19.5 ± 2.7 (mean ± SD) years and 63% identified as female (Table 2). Most survey respondents were studying full time (65%) and the highest proportion were living out of their family home (44%) (Table 2). Most had attended *headspace* between 1 and 6 times (56%), and more than half had received a mental health diagnosis (54%) (Table 2). Four young people completed individual interviews and four participated in a focus group. Of these participants, 75% identified as female (*n* = 6); and age ranged from 20 to 25 years. Four main themes were identified (i) Dietary behaviors, knowledge, and barriers to healthy eating (ii) Behaviors, knowledge, and barriers to physical activity; (iii) The perceived relationship between nutrition and physical activity with mental health; and (iv) The role of youth mental health services in supporting nutrition and physical activity. Survey, interview and focus group findings are presented under each theme.

**Table 2:** Demographic information and frequency of mental health service use in the study sample of young people (*n* = 48)

Age (years)	Number	Percentage
15–17	15	31%
18–21	16	33%
22–25	12	25%
Not answered	5	11%
**Gender**		
Female	30	63%
Male	12	25%
Other	2	4%
Not answered	4	8%
**Aboriginal/Torres Strait Islander**		
Yes	5	11%
**Living Situation**		
Living out of home by myself or with others	21	44%
Living at home with both parents/stepparents	16	33%
Living at home with 1 parent	3	6%
Short-term or unstable accommodation	2	4%
Homeless/sleeping rough	1	2%
Not answered	5	11%
**Suburb/Town**		
Launceston area	35	73%
Outside Launceston	6	12%
Not answered	7	15%
**Work/Study Status**		
School/TAFE/Other education	31	65%
Full-time work	7	15%
Part-time work	18	38%
Receive payments from Centrelink	16	33%
Home/parenting duties	2	4%
**Number of contacts with** ***headspace*** **in the past 12 months**		
1–6 sessions	27	56%
7–11	5	11%
12+	10	21%
Not answered	6	12%
**Mental health diagnosis**		
Yes	26	54%
**If yes, diagnosis**		
Anxiety	18	38%
Depression	22	46%
Other: including Borderline Personality Disorder; ADHD, Autism Spectrum Disorder; eating disorder; Bipolar; Postpartum Depression; Complex PTSD	11	23%

### Dietary behaviors, knowledge, and barriers to healthy eating

Most survey respondents did not meet the Australian government recommendations for fruit (81%) and vegetable consumption (92%) (Table 3) in line with their age group. Additionally, most respondents (67%) skipped breakfast on one or more days during the week. While the majority (81%) of survey respondents consumed water on a daily basis, only 38% consumed it several times per day. Most survey respondents consumed drinks with added sugars (58%) and takeaway foods (75%), once a week or more. Most respondents (69%) correctly identified that they should consume less drinks with added sugar, however a minority of survey respondents (*n* = 2) who consumed drinks with added sugar more than once per day incorrectly reported that they should continue to consume this amount. The majority of the respondents (71%) correctly identified they should consume less take-away foods.

**Table 3:** Proportion (n (%)) of survey respondents’ consumption of fruit, vegetables, breakfast, sugar sweetened beverages and takeaway foods

	Category	n	%
Fruit intake	Meeting recommendations	9	18.8%
	Not meeting recommendations	39	81.3%
Vegetable Intake	Meeting recommendations	4	8.3%
	Not meeting recommendations	44	91.7%
Breakfast Intake	Not eating daily	32	66.7%
	Eating daily	16	33.3%
Water Intake	Multiple times per day	18	37.5%
	Once a day or less	30	62.5%
Sugar sweetened beverages intake	Less than once a week	20	41.7%
	Weekly or more	28	58.3%
Takeaway food intake	Less than once a week	12	25.0%
	More than once a week	36	75.0%

For nutrition knowledge, most (77%) survey respondents identified that monitoring food consumption behavior could help to maintain a healthy weight, but less than half identified not eating while watching the TV (40%) and reading food labels (27%) as behaviors that could help to maintain a healthy weight, and were identified as helpful strategies according to the General Nutrition Knowledge Questionnaire [42]. More than a third of survey respondents reported that snacking throughout the day (37%) and taking nutritional supplements (77%) was a strategy to maintain a healthy weight, which was incorrect according to the General Nutrition Knowledge Questionnaire [42]. All participants who took part in an interview and/or focus group were able to describe that fruit and vegetables form part of a healthy diet. Some of these participants described having the nutrition to support energy needs throughout the day and were able to provide examples of healthy foods including fruit, vegetables, protein and grains. Most participants were able to describe a “pyramid”, “plate”, or “2 and 5 campaign”, however they were unable to provide more detail about current national nutrition guidelines.

*It means a good balanced diet and making sure that you’re not having too much of one thing and not enough of another. So just making sure you’ve got a good balance of fruit and veg, and sweets and stuff like that, but in moderation –* Interview Participant 3

**Fig. 2:**
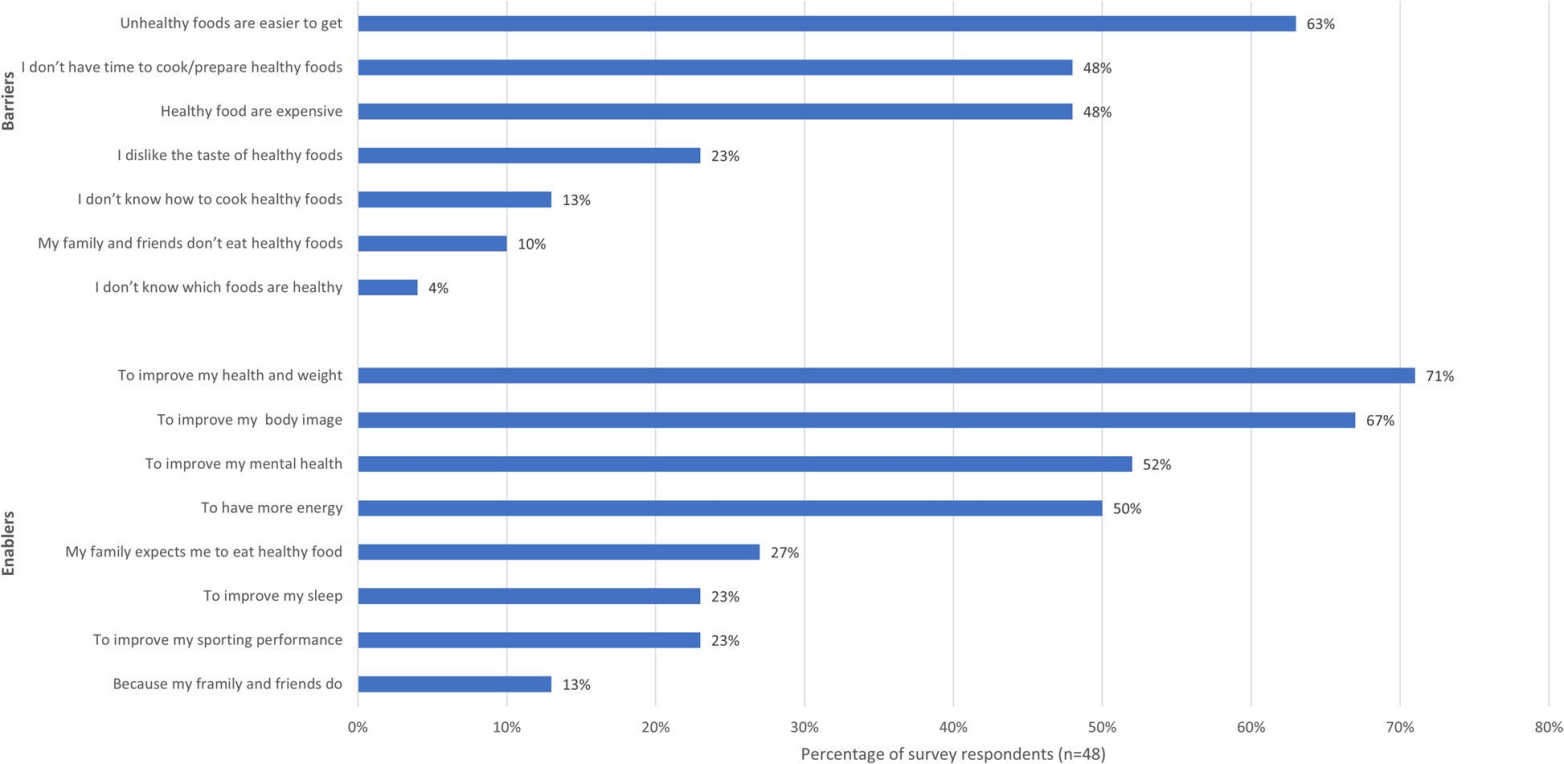
Percentage of survey respondents who indicated agreement with a series of statements about the main barriers and enablers to healthy eating

Figure 2 identifies the main barriers and enablers to healthy eating identified by survey respondents. Food access, time and expense of food were the primary barriers, with knowledge of cooking and healthy foods less commonly reported. Similar barriers were noted through the interview and focus group, with the main reported barriers to healthy eating being accessibility or convenience of fast food, perceived cost of healthy foods, and no time to prepare meals.

*Often eating healthily is more difficult than eating junk food. It’s very easy to just walk past KFC or McDonalds or Dominoes or wherever and to just buy readymade bit of takeaway but to eat healthily, it takes more work* – Interview Participant 4

The primary enabling factors identified through the survey for healthy eating as shown in Fig. 2 were to improve health and weight, body image and mental health. In addition, during interview and focus group, the most frequently reported motivator was social support:

*I definitely think that the people around you make an influence about what you eat. So, my partner likes to eat quite healthily, he does a lot of meal prep, so then that encourages me to do the same.* – Interview Participant 3

### Behaviors, knowledge, and barriers to physical activity

The majority of survey respondents (79%) identified walking as their main form of physical activity. A smaller proportion of survey participants identified going to the gym (33%), running (27%) and organized sport i.e. football, netball (23%) as additional forms of physical activity they participated in. Other forms of physical activity identified by individual survey respondents included dancing, swimming, bike riding/motor cross, karate and yoga (37%).

For survey respondents aged between 15 and 17, approximately a quarter (27%) met the Australian government’s physical activity requirements for their age group, and most of these survey respondents correctly identified they were doing enough physical activity. Of the survey respondents that were identified as adults, 36% (10 survey respondents) met the Australian government’s physical activity requirements for adults. Four out of the 10 survey respondents identified as adults were able to correctly identify they were already meeting the physical activity requirements. The 5 remaining survey respondents did not provide their ages; however, none met the requirements for either children or adults.

**Fig. 3:**
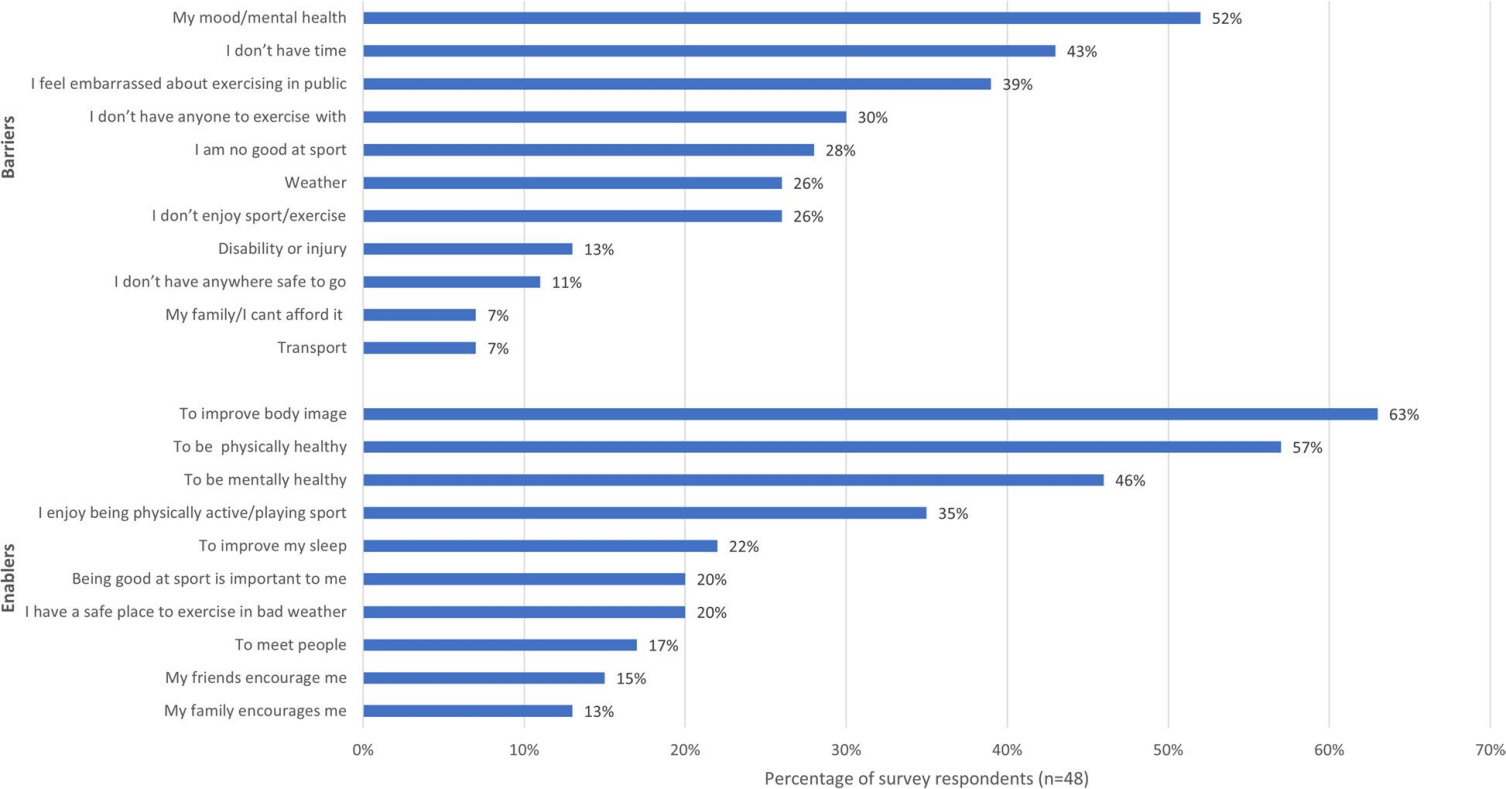
Percentage of survey respondents who indicated agreement with a series of statements about the main barriers and enablers to physical activity

Figure 3 identifies the main barriers and enablers to physical activity identified by survey respondents. Main barriers of mood, time and embarrassment of exercising in public were identified through the survey. Conversely, no time, negative experiences during childhood, and low skills and confidence were identified as barriers through the interviews/focus group.

*I don’t necessarily do as much as I should or to the extent where maybe I will only go once a week rather than once a day. I would like to be able to go once a day but it’s definitely something that I struggle to find time to fit in* – Interview Participant 2

The main enablers for physical activity for survey respondents included improved body image, to be physically healthy, and to be mentally healthy (Fig. 3). Social support was the main enabler identified through both the interviews and focus group.

*We both go to the gym … We go with a few of our friends. And it would make it.. It’s so fun … We sort of game-ify it* – Focus Group Participant

### Preferred providers and sources of nutrition and physical activity support

**Fig. 4:**
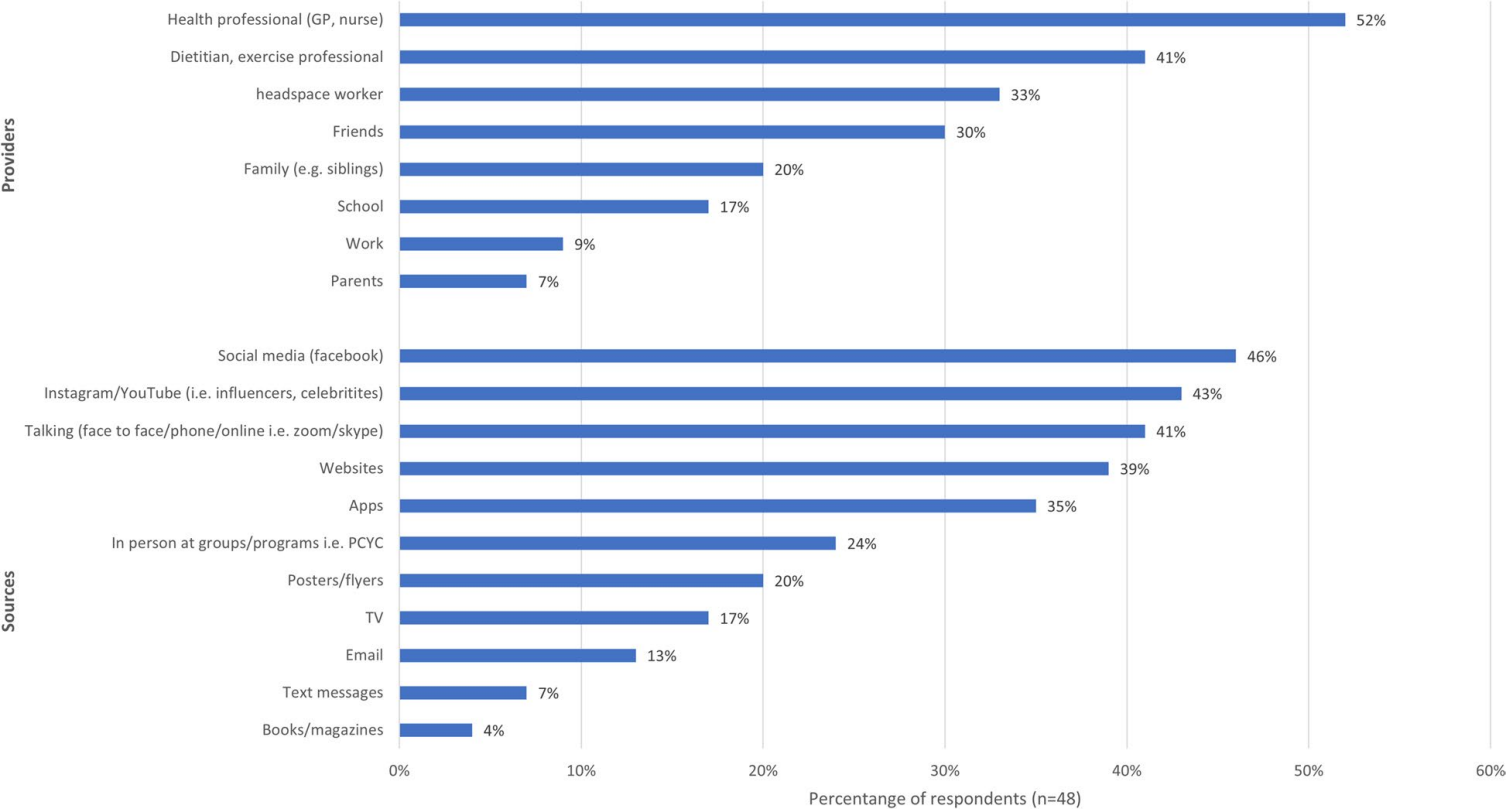
The proportion of survey respondents indicating the preferred provider and the source of receiving nutrition and physical activity information and support in a sample of young people attending a youth mental health service

Figure 4 indicates who and where/how survey respondents would like to receive nutrition and physical activity information from. Additionally, interview and focus group participants described school as their main source of information for both healthy eating and physical activity behaviors. The internet (social media i.e. Facebook, Instagram) were also identified as a source of information for healthy eating. Most respondents expressed concern about the confusing amount of information on the internet and challenges of knowing who to believe, particularly when paid advertising from “influencers” overwhelmed their social media feed.

*General high school level education is probably the most that I've had about food without being involved in anything cooking. I think there is not enough to extend that knowledge, especially when you have things like fad diets all coming into socials – like … keto, paleo … – it’s only bad stuff that I would see on my social media or hear from people –* Interview Participant 2

The perceived relationship between nutrition and physical activity with mental health.

Two thirds of survey respondents (66%) reported that they considered good nutrition was important for managing mental health (Table 4), and 61% reported that physical activity was important for managing mental health (Table 4). The relationship between both healthy eating and physical activity and mental health were reported as enabling factors by approximately half of survey respondents (Figs. 2 and 3). During interviews and the focus group, participants strongly recognized the bidirectional link between healthy eating, physical activity and their mental health. Participants were aware that physical activity improved their mood and reduced stress. Participants were also aware that poor eating behaviors (e.g., skipping meals), poor food quality or consuming unhealthy foods directly impacted their mood. Some were aware that certain foods impacted their mood but were unaware of exactly how this occurred. One participant particularly noticed the negative impact of moving out of home on their healthy eating, physical activity and mental health:

*… when I’m feeling down, I tend to go towards not so good foods, and it never really makes me feel any better. It then leads to stuff like fatigue, because you’re not getting the right sort of fuels in your body, and then potential weight gain … , which can then have a negative impact on your mental health … So, eating the right sort of foods like your fruit and veggie gives you the energy, and then you can go out and go for your walk and build up, get your serotonin levels going. And that helps –* Interview Participant 3

**Table 4:** Proportion (n (%)) of survey respondents’ percieved importance of nutrition and physical activity for managing mental health

		n	%
The perceived importance of nutrition for managing mental health	Not important	7	14.9%
	Neutral	9	19.1%
	Important	31	66.0%
The perceived importance of physical activity for managing mental health	Not important	8	18.1%
	Neutral	9	20.4%
	Important	27	61.3%

### The role of youth mental health services in supporting nutrition and physical activity

Of the survey respondents, only 33% reported that a source of nutrition and physical activity information and support should come from *headspace* (Fig. 4). However, all participants in the interviews and focus group expressed that *headspace* could have a stronger role in supporting healthy eating and physical activity. Most participants felt it would be helpful for *headspace* staff to be proactive in raising the topics as part of sessions, rather than the young person having to raise the topic. Participants felt that *headspace* staff could offer useful resources, facilitate referrals to health professionals, provide information on sports options and offer a personal link to these.

*I had my therapist appointment this morning and we were talking about how my unhealthy eating was impacting me. It would have been awesome if she had a resource then that she could be like, “Actually, I know someone perfect to talk to you about that; here’s who you can talk to, they are going to be able to help you with this.” That sort of thing –*Interview Participant 1

When participants reflected on their past experiences of accessing support at *headspace,* most acknowledged that receptivity to conversations initiated about healthy eating and physical activity during sessions would vary due to individual needs, maturity levels, priorities for attending sessions and motivation. One reflected they had had “general” conversations about healthy eating during headspace sessions, whilst another described a significant improvement in healthy eating and physical activity due to attendance at *headspace* sessions.

*My food knowledge has drastically increased and through going to headspace and talking to my counsellor there –* Interview Participant 2

Most participants expressed enthusiasm for the idea of accessing a dietitian or exercise physiologist through *headspace*, but were concerned about the barriers to this service, such as cost.

## Discussion

This novel study comprehensively assessed nutrition and physical activity behaviors and knowledge, the main barriers and enablers to achieving recommendations and the preferred sources of support among a sample of young people attending a mental health service in regional Australia. Our results suggest that most young people in our study were knowledgeable about how their behavior compared to Australian Government diet and physical activity recommendations and understood the role of diet and physical activity for maintaining their mental health. However, participants reported poor nutrition and physical activity behaviors and experienced substantial barriers towards achieving recommendations. While support for improving nutrition and physical activity behaviors from reputable providers including health professionals was seen as a priority, most participants indicated they wanted to receive these messages through social media channels. Our results point to a clear opportunity to improve nutrition and physical activity behaviors in this vulnerable group, which could enhance both mental and physical health outcomes across their lifespan [46].

Our results indicate that a knowledge/behavior gap surrounding nutrition and physical activity appears in this sample of young people with mental health issues. The poor dietary behaviors in our study sample are similar to results from previous studies in a large sample of young people in Australia who consumed very low levels of fruit and vegetables [47] and had a high consumption of takeaway foods and drinks with added sugar [48]. Critically, these foods may be displacing nutrient-rich alternatives if consumed too frequently, leading to poor outcomes for both physical and mental health [48]. In our study, most participants reported either skipping or not eating breakfast on a regular basis. This was substantially higher than recent studies that show only 8% of Australian youth skipped breakfast [49] and may identify a significant gap in dietary behaviors in this particularly vulnerable group of youths. Indeed, a greater frequency of skipping breakfast is associated with a higher probability of experiencing depressive symptoms [50], and young people who consume breakfast have better mental health [51], indicating that this is a potential area of action for this group.

Further in our study, few participants met the recommendations for physical activity, which is not dissimilar to the levels of physical inactivity in young people nationally which show 18% of 12–17 year old and 6–22% of 15–17 year olds are not sufficiently active [52]. Walking was the most commonly reported type of physical activity, which is dissimilar to the organized sport preferences of younger Australian children [53]. Walking is generally considered to be a low-intensity physical activity, which has shown to lack the benefits of moderate or vigorous intensity physical activity for supporting cognitive and mental health measures in young people [54]. Thus, further integration of different types of physical activity (inclusive of moderate and/or vigorous intensity tasks) into the treatment and support for young people with mental health issues as an early intervention strategy would be important for supporting physical and mental health outcomes for this group [55].

In our study, most participants were aware of how their diet and physical activity compared with public health recommendations, however, substantial barriers in achieving positive behavior changes were reported. Time and expense ‘costs’ associated with healthy eating and physical activity were considerable factors limiting positive behaviors by participants in our study, with most reporting unhealthy food is easier to access. While Australian data shows that healthy diets can be more affordable than current (unhealthy) diets [56], research also shows that healthy diets are still unaffordable for some low-socioeconomic groups [57]. Given more than a third of our survey sample were receiving government financial assistance, financial and physical access to healthy food and opportunities appears to be a major issue in this group. The paradoxical result of mood being reported as an enabling factor and a barrier has also been reported in adult populations, with previous research indicating that stress and depression limits physical activity participation in more than 60% of adults with severe mental illness [58]. Our results highlight that in addition to challenges to changing health behaviors in young people in general, for those with poor mental health, barriers are amplified. Our results are similar to other research that shows improving body image, physical health and weight were amongst the top enabling factors for improving diet and physical activity in young people [59]. In one study in young men, those reporting they experienced greater stress were significantly more likely to rank mental health as a key motivator compared to those experiencing lesser symptoms of stress [59]. Given the relationship between all these factors and mental health in young people, strategies that improve physical activity and nutrition could promote positive weight and body image outcomes in addition to highlighting the benefits to mental wellbeing could be applied in this group. Such strategies, based on self-determination theory, that focus on promoting autonomous self-regulatory behavior have been previously demonstrated as effective [60].

The young people in our study reported wanting to receive health-related nutrition and physical activity support from various, qualified health professionals. This aligns with research that suggests that young people desire reliable and quality health care information [61], however in our study, young people commonly reported wanting to receive this information through social media channels. This finding was also confirmed in other Australian research, which was related to the assumed anonymity and privacy that social media offers [62], in addition to online health information being readily available [63]. Young people commonly search health related information through online sources [64]. In our study, the interview and focus group participants expressed concern about how health information can be confusing and misleading when accessed online, particularly when paid advertisements filled their social media. As such, they expressed a desire for trusted health professionals to deliver the information, or to help them make sense of the information they find online. In addition, young people reported that talking face-to-face was important to them, highlighting that conversations with trusted providers, such as mental health services remain relevant to young people. Given how widely social media is accessed by young Australians, it is important to explore further how this could be an effective tool for nutrition and physical activity support in young people with mental health issues. Peer-to-peer support using digital technologies is a preferred method for young people with mental health concerns to promote positive health behaviors [65]. Congruent with self determination theory [66], previous health promotion programs have demonstrated the importance of social connectedness and peer support in generating positive behavior change outcomes.

In our study, it was identified that the mental health service was among the top three preferred providers of nutrition and physical activity information, and that some conversations towards diet and physical activity had occurred already for some young people during sessions at *headspace*. However, given the priority focus on mental health, time pressure and lack of specialization of mental health counsellors in this field it is likely that these were limited to general comments about nutrition and physical activity for wellbeing, and referrals to professional services. While some preliminary evidence of the acceptability of an exercise program in young people attending a youth mental health service has been published [67], embedding this approach into standard psychological treatment for young people should be considered. Improving access for young people to receive nutrition and physical activity interventions may promote their knowledge and confidence, addressing the competency aspect of behavior change [66]. Integrating support for improving nutrition and physical activity behaviors into current counselling sessions may also be preferable to specialized programs, as it has previously been shown that consumer financial status and other responsibilities can influence program access and attendance in people with mental health issues [68].

### Strengths and limitations

This study has a number of strengths including presenting detailed information about a range of factors impacting nutrition and physical activity behaviors in young people attending a youth mental health service that can be used to directly inform strategies to support young people. Despite this, limitations of this study are recognised. First, the study is limited by a small sample size due to recruitment challenges and project timeline constraints, which may reduce the power of the study and increase the margin of error. The convenience sampling utilized limits the transferability of the research. Additionally, the use of paid advertisement on social media to increase response rates means that researchers cannot be certain that all participants met the eligibility criteria, as this was self-reported. The use of the three screening questions for consent impacted the number of young people accessing the survey. In total, only 58 of 141 (41%) who attempted to access the survey were able to answer the screening questions correctly (despite efforts to provide multiple sources of study information in varying media formats). It is also possible that consent questions were not answered correctly due to low literacy levels, participants not reading/listening to the study information or the study information not being engaging enough for the needs of the cohort. Some survey questions were adapted from validated measures, such as The International Physical Activity Questionnaire [69], however most survey questions developed for the purpose of this study to be as simple as possible for this population group, regardless of validation, which might contribute to measurement error in this study. While our study selected appropriate, low literacy questions for screening participants for dietary behaviors to increase engagement, a more comprehensive assessment of diet using validated tools, such as a food frequency questionnaire, should be a priority for future research to more accurately quantify dietary patterns in this at-risk group. Finally, the study was conducted throughout the COVID-19 pandemic, resulting in delays and limited access to the mental health service at the height of lockdowns. During the study, Tasmania had not had any transmission of COVID. However, it is possible these contextual factors may still have influenced the findings.

## Conclusions

Our study, conducted in a sample of young people attending a regional youth mental health service, indicates that most young people are knowledgeable about how their behaviors compare to Australian government nutrition and physical activity recommendations and understand their role in maintaining their mental health. However, both diet and physical activity behaviors in this group were poor and there are substantial barriers to achieving these recommendations including poor mental health. Our results indicate that young people would prefer support for reputable information and advice to be communicated through social media. Given the results from this study, which highlights the large scale of poor diet and physical activity behaviors in youth mental health settings, it can be recommended that health professionals be commissioned to either produce or review materials to ensure young people have access to credible and accurate information and support. Understanding the complexities of behavior change from a self-determination perspective may also enhance the efficacy of interventions within the youth mental health context. There is an increasing awareness of the importance of dietitians and exercise physiologists in standard care. Further research is still required into how mental health care plans can be extended beyond just psychologists to also include appointments with other allied health professionals, given the increasing importance of multidisciplinary support in the maintenance of good mental health in young people. Additionally, future research should focus on how to best implement lifestyle interventions that improve nutrition and physical activity behaviors to support mental health outcomes in young people attending mental health services.

## Supplementary Information

**Additional file 1.**

**Additional file 2.**

## Data Availability

The datasets generated and/or analyzed during the current study are not publicly available due ongoing data analysis but are available from the corresponding author on reasonable request.
