# Supplementary material for: The knowledge, barriers and opportunities to improve nutrition and physical activity amongst young people attending an Australian youth mental health service: a mixed-methods study
Source: BMC Health Serv Res. 2022 Jun 17;22:789. doi: 10.1186/s12913-022-08182-0 (PMC9205652; doi:10.1186/s12913-022-08182-0)
Supplement: Supplementary file 1 — Additional file 1. [file 12913_2022_8182_MOESM1_ESM.pdf]

Understanding Healthy Eating and Physical Activity Beliefs, Behaviours and Needs of Young People Accessing Headspace Launceston

Hello thanks for doing our survey! If you would like someone to help you to fill the survey in please call Heather on 0411 922 723. Just remember this survey is anonymous so no one will know your answers. Please click on the links below to read or listen to the Information Sheet.

At the end of the survey you will be invited to take part in a one-to-one interview. If you prefer just to do the interview, please contact Heather on 0411 922 723 or at Heather.Bridgman@utas.edu.au.

If at any time you want to stop the survey just close the page or if you feel upset in any way please contact headspace on 6335 3100.

Participant Information Sheet (/upload/surveys/884437/files/headspace-heapa-info-sheet.pdf)

Information Video (https://www.utas.edu.au/rural-health/projects-and-activities/projects/headspace-survey)

If you would like to take part, click [Next].

There are 35 questions in this survey.

Consent

Are you currently under a child protection/guardianship order (living away from your parent/s)? *

Please choose **only one** of the following:

- ◯ Yes
- ◯ No

True or False

Please answer these two questions before starting the survey.

Tick which of these are true? *

Only answer this question if the following conditions are met:
Answer was 'No' at question '1 [surveyFilter]' (Are you currently under a child protection/guardianship order (living away from your parent/s)?)

❗ Choose one of the following answers
Please choose **only one** of the following:

- ◯ Headspace will know if I’ve done this survey
- ◯ No one will know I’ve done this survey
- ◯ Not sure

Tick which of these are true? *

Only answer this question if the following conditions are met:
Answer was 'No' at question '1 [surveyFilter]' (Are you currently under a child protection/guardianship order (living away from your parent/s)?)

❶ Choose one of the following answers
Please choose **only one** of the following:

- ◯ If I start the survey I have to finish it
- ◯ If I start the survey I can stop at anytime and no one will know
- ◯ Not sure

Food

First we would like to ask you some questions about food

How many serves of fruit do you USUALLY eat each day (including all fresh, dried, frozen, and tinned fruit)?

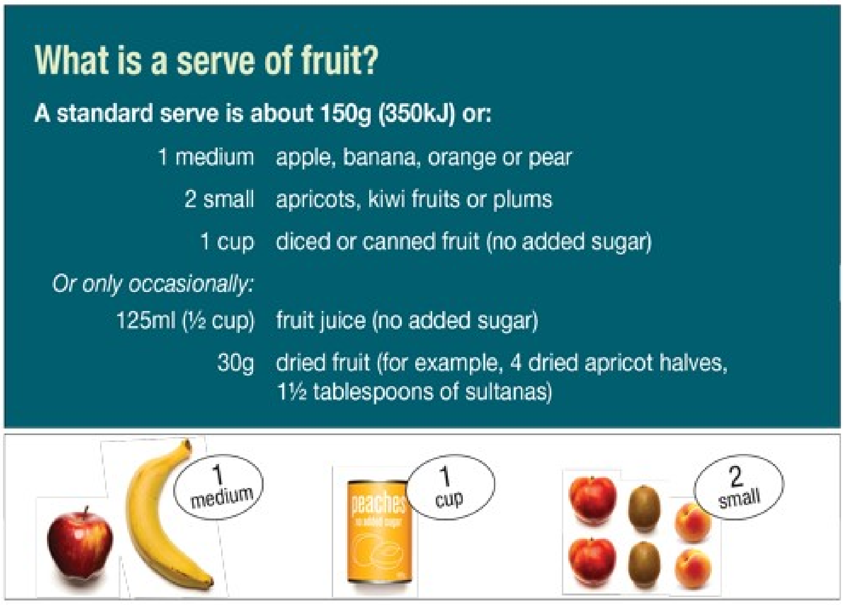

Source: https://www.eatforhealth.gov.au/

Only answer this question if the following conditions are met:
Answer was 'No' at question '1 [surveyFilter]' (Are you currently under a child protection/guardianship order (living away from your parent/s)?) *and* Answer was 'No one will know I've done this survey' at question '2 [filter1]' (Tick which of these are true?) *and* Answer was 'If I start the survey I can stop at anytime and no one will know' at question '3 [filter2]' (Tick which of these are true?)

❗ Choose one of the following answers
Please choose **only one** of the following:

◯ I don't eat fruit every day

◯ 1 serve or less

◯ 2 serves or more

How many serves of vegetables do you USUALLY eat each day (including fresh, frozen and tinned vegetables)?

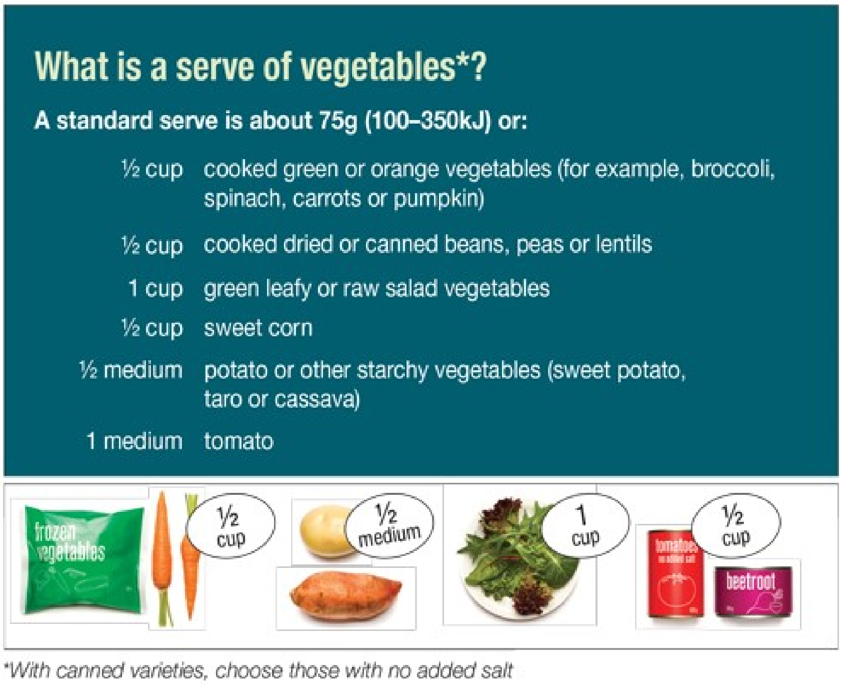

Source: https://www.eatforhealth.gov.au/

Only answer this question if the following conditions are met:
Answer was 'No' at question '1 [surveyFilter]' (Are you currently under a child protection/guardianship order (living away from your parent/s)?) *and* Answer was 'No one will know I’ve done this survey' at question '2 [filter1]' (Tick which of these are true?) *and* Answer was 'If I start the survey I can stop at anytime and no one will know' at question '3 [filter2]' (Tick which of these are true?)

❗ Choose one of the following answers
Please choose **only one** of the following:

◯ 1 serve or less

◯ 2, 3 or 4 serves

◯ 5 serves or more

How often do you USUALLY eat these foods and drinks (tick one option per line)?

Only answer this question if the following conditions are met:
Answer was 'No' at question '1 [surveyFilter]' (Are you currently under a child protection/guardianship order (living away from your parent/s)?) *and* Answer was 'No one will know I’ve done this survey' at question '2 [filter1]' (Tick which of these are true?) *and* Answer was 'If I start the survey I can stop at anytime and no one will know' at question '3 [filter2]' (Tick which of these are true?)

Please choose the appropriate response for each item:

	More than once a day	Daily	A couple of days a week	Weekly	Less than once a week	Never or rarely
**Drinks with added sugar (such as energy drinks, soft drinks, cordials and sports drinks)**	◯	◯	◯	◯	◯	◯
**Take-away foods (such as burgers, pizza, sausage rolls, or hot chips)**	◯	◯	◯	◯	◯	◯
**Water (including tap and bottled water)**	◯	◯	◯	◯	◯	◯
**Breakfast**	◯	◯	◯	◯	◯	◯

Do you think you should eat more, the same amount or less of the following foods (tick one box per line)

Only answer this question if the following conditions are met:
Answer was 'No' at question '1 [surveyFilter]' (Are you currently under a child protection/guardianship order (living away from your parent/s)?) *and* Answer was 'No one will know I’ve done this survey' at question '2 [filter1]' (Tick which of these are true?) *and* Answer was 'If I start the survey I can stop at anytime and no one will know' at question '3 [filter2]' (Tick which of these are true?)

Please choose the appropriate response for each item:

	**More**	**Same**	**Less**	**Not Sure**
**Fruit**	◯	◯	◯	◯
**Vegetables**	◯	◯	◯	◯
**Drinks with added sugar**	◯	◯	◯	◯
**Take-away foods**	◯	◯	◯	◯
**Water**	◯	◯	◯	◯
**Breakfast**	◯	◯	◯	◯

Which of these options can help YOU have a healthy weight? (tick one box per line)

Only answer this question if the following conditions are met:
Answer was 'No' at question '1 [surveyFilter]' (Are you currently under a child protection/guardianship order (living away from your parent/s)?) *and* Answer was 'No one will know I’ve done this survey' at question '2 [filter1]' (Tick which of these are true?) *and* Answer was 'If I start the survey I can stop at anytime and no one will know' at question '3 [filter2]' (Tick which of these are true?)

Please choose the appropriate response for each item:

	Yes	No	Not Sure
**Not eating while watching TV**	◯	◯	◯
**Reading food labels**	◯	◯	◯
**Taking nutritional supplements**	◯	◯	◯
**Monitoring how much you eat**	◯	◯	◯
**Snacking throughout the day**	◯	◯	◯

How important is healthy food to you in managing your mental health?

Only answer this question if the following conditions are met:
Answer was 'No' at question '1 [surveyFilter]' (Are you currently under a child protection/guardianship order (living away from your parent/s)?) *and* Answer was 'No one will know I’ve done this survey' at question '2 [filter1]' (Tick which of these are true?) *and* Answer was 'If I start the survey I can stop at anytime and no one will know' at question '3 [filter2]' (Tick which of these are true?)

Please choose **only one** of the following:

◯ 1
◯ 2
◯ 3
◯ 4
◯ 5

⋆ [Not very important]

⋆⋆ [Not important]

⋆⋆⋆ [Don't know]

⋆⋆⋆⋆ [Important]

⋆⋆⋆⋆⋆ [Very Important]

Over the past year did you or your family ever run out of food and not have money to buy more?

(remember this is an anonymous survey)

Only answer this question if the following conditions are met:
Answer was 'No' at question '1 [surveyFilter]' (Are you currently under a child protection/guardianship order (living away from your parent/s)?) *and* Answer was 'No one will know I’ve done this survey' at question '2 [filter1]' (Tick which of these are true?) *and* Answer was 'If I start the survey I can stop at anytime and no one will know' at question '3 [filter2]' (Tick which of these are true?)

❗ Choose one of the following answers
Please choose **only one** of the following:

◯ Yes
◯ No
◯ I don't know

What stops you from eating healthy food?

Only answer this question if the following conditions are met:
Answer was 'No' at question '1 [surveyFilter]' (Are you currently under a child protection/guardianship order (living away from your parent/s)?) *and* Answer was 'No one will know I’ve done this survey' at question '2 [filter1]' (Tick which of these are true?) *and* Answer was 'If I start the survey I can stop at anytime and no one will know' at question '3 [filter2]' (Tick which of these are true?)

❗ Check all that apply
Please choose **all** that apply:

- ☐ Unhealthy food is easier to get
- ☐ I don’t have time to cook/prepare healthy foods
- ☐ healthy foods are expensive
- ☐ I don’t know how to cook healthy foods
- ☐ I dislike the taste of healthy foods
- ☐ My friends and family don’t eat healthy food
- ☐ I don’t know which foods are healthy
- ☐ Nothing
- ☐ Other: ____________

What motivates you to choose healthy foods?

Only answer this question if the following conditions are met:
Answer was 'No' at question '1 [surveyFilter]' (Are you currently under a child protection/guardianship order (living away from your parent/s)?) *and* Answer was 'No one will know I’ve done this survey' at question '2 [filter1]' (Tick which of these are true?) *and* Answer was 'If I start the survey I can stop at anytime and no one will know' at question '3 [filter2]' (Tick which of these are true?)

❗ Check all that apply
Please choose **all** that apply:

- ☐ To improve my health and weight
- ☐ To improve my body image
- ☐ To have more energy
- ☐ To improve my sporting performance,
- ☐ To improve my mental health
- ☐ To improve my sleep
- ☐ Because my friends and family eat healthy food
- ☐ My family expects me to eat healthy food
- ☐ Nothing
- ☐ Other: ____________

Physical Activity and Sports

Now we would Like to ask you some questions about physical activity and sports.

What physical activity/sports do you USUALLY do?

Only answer this question if the following conditions are met:
Answer was 'No' at question '1 [surveyFilter]' (Are you currently under a child protection/guardianship order (living away from your parent/s)?) *and* Answer was 'No one will know I've done this survey' at question '2 [filter1]' (Tick which of these are true?) *and* Answer was 'If I start the survey I can stop at anytime and no one will know' at question '3 [filter2]' (Tick which of these are true?)

❗ Check all that apply
Please choose **all** that apply:

- ☐ Walking
- ☐ Running
- ☐ Swimming
- ☐ Gym
- ☐ Dancing
- ☐ Yoga
- ☐ PDHPE lessons
- ☐ Bike riding
- ☐ Organised sport e.g football, netball
- ☐ Other: ______

How many times a week would you USUALLY do light physical activity such as (e.g casual walking, washing the dishes or yoga)?

Only answer this question if the following conditions are met:
Answer was 'No' at question '1 [surveyFilter]' (Are you currently under a child protection/guardianship order (living away from your parent/s)?) *and* Answer was 'No one will know I've done this survey' at question '2 [filter1]' (Tick which of these are true?) *and* Answer was 'If I start the survey I can stop at anytime and no one will know' at question '3 [filter2]' (Tick which of these are true?)

❗ Choose one of the following answers
Please choose **only one** of the following:

- ◯ I don't do light physical activity
- ◯ Less than once a week
- ◯ Weekly
- ◯ A couple of days a week
- ◯ Daily
- ◯ More than once a day

How long would you USUALLY do light physical activity when you do it?

Only answer this question if the following conditions are met:
Answer was 'No' at question '1 [surveyFilter]' (Are you currently under a child protection/guardianship order (living away from your parent/s)?) *and* Answer was 'No one will know I’ve done this survey' at question '2 [filter1]' (Tick which of these are true?) *and* Answer was 'If I start the survey I can stop at anytime and no one will know' at question '3 [filter2]' (Tick which of these are true?)

❗ Choose one of the following answers
Please choose **only one** of the following:

- ◯ Less than 15 minutes
- ◯ Between 15 and 30 minutes
- ◯ Between 30 and 60 minutes
- ◯ Longer than 60 minutes
- ◯ None

How many times a week would you USUALLY do moderate physical activity (e.g. brisk walking or dancing)?

Only answer this question if the following conditions are met:
Answer was 'No' at question '1 [surveyFilter]' (Are you currently under a child protection/guardianship order (living away from your parent/s)?) *and* Answer was 'No one will know I’ve done this survey' at question '2 [filter1]' (Tick which of these are true?) *and* Answer was 'If I start the survey I can stop at anytime and no one will know' at question '3 [filter2]' (Tick which of these are true?)

❗ Choose one of the following answers
Please choose **only one** of the following:

- ◯ I don’t do moderate physical activity
- ◯ Less than once a week
- ◯ Weekly
- ◯ A couple of days a week
- ◯ Daily
- ◯ More than once a day

How long would you USUALLY do moderate physical activity when you do it?

Only answer this question if the following conditions are met:
Answer was 'No' at question '1 [surveyFilter]' (Are you currently under a child protection/guardianship order (living away from your parent/s)?) *and* Answer was 'No one will know I’ve done this survey' at question '2 [filter1]' (Tick which of these are true?) *and* Answer was 'If I start the survey I can stop at anytime and no one will know' at question '3 [filter2]' (Tick which of these are true?)

❗ Choose one of the following answers
Please choose **only one** of the following:

- ◯ Less than 15 minutes
- ◯ Between 15 and 30 minutes
- ◯ Between 30 and 60 minutes
- ◯ Longer than 60 minutes
- ◯ None

How many times a week would you USUALLY do hard physical activity (e.g. running, football, group exercise classes or weight training)?

Only answer this question if the following conditions are met:
Answer was 'No' at question '1 [surveyFilter]' (Are you currently under a child protection/guardianship order (living away from your parent/s)?) *and* Answer was 'No one will know I’ve done this survey' at question '2 [filter1]' (Tick which of these are true?) *and* Answer was 'If I start the survey I can stop at anytime and no one will know' at question '3 [filter2]' (Tick which of these are true?)

❗ Choose one of the following answers
Please choose **only one** of the following:

- ◯ I don’t do high intensity physical activity
- ◯ Less than once a week
- ◯ Weekly
- ◯ A couple of days a week
- ◯ Daily
- ◯ More than once a day

How long would you USUALLY do hard physical activity when you do it?

Only answer this question if the following conditions are met:
Answer was 'No' at question '1 [surveyFilter]' (Are you currently under a child protection/guardianship order (living away from your parent/s)?) *and* Answer was 'No one will know I’ve done this survey' at question '2 [filter1]' (Tick which of these are true?) *and* Answer was 'If I start the survey I can stop at anytime and no one will know' at question '3 [filter2]' (Tick which of these are true?)

❗ Choose one of the following answers
Please choose **only one** of the following:

- ◯ Less than 15 minutes
- ◯ Between 15 and 30 minutes
- ◯ Between 30 and 60 minutes
- ◯ Longer than 60 minutes
- ◯ None

Do you think you should do more, the same amount or less of the following activities (tick one box per line)

Only answer this question if the following conditions are met:
Answer was 'No' at question '1 [surveyFilter]' (Are you currently under a child protection/guardianship order (living away from your parent/s)?) *and* Answer was 'No one will know I’ve done this survey' at question '2 [filter1]' (Tick which of these are true?) *and* Answer was 'If I start the survey I can stop at anytime and no one will know' at question '3 [filter2]' (Tick which of these are true?)

Please choose the appropriate response for each item:

	More	Same	Less	Not sure
**Light physical activity**	◯	◯	◯	◯
**Moderate physical activity**	◯	◯	◯	◯
**Hard physical activity**	◯	◯	◯	◯

How important is physical activity to you in managing your mental health?

Only answer this question if the following conditions are met:
Answer was 'No' at question '1 [surveyFilter]' (Are you currently under a child protection/guardianship order (living away from your parent/s)?) *and* Answer was 'No one will know I've done this survey' at question '2 [filter1]' (Tick which of these are true?) *and* Answer was 'If I start the survey I can stop at anytime and no one will know' at question '3 [filter2]' (Tick which of these are true?)

Please choose **only one** of the following:

◯ 1

◯ 2

◯ 3

◯ 4

◯ 5

⋆ [Not very important]

⋆⋆ [Not important]

⋆⋆⋆ [Don't know]

⋆⋆⋆⋆ [Important]

⋆⋆⋆⋆⋆ [Very Important]

What stops you from participating in physical activity and/or sports?

Only answer this question if the following conditions are met:
Answer was 'No' at question '1 [surveyFilter]' (Are you currently under a child protection/guardianship order (living away from your parent/s)?) *and* Answer was 'No one will know I’ve done this survey' at question '2 [filter1]' (Tick which of these are true?) *and* Answer was 'If I start the survey I can stop at anytime and no one will know' at question '3 [filter2]' (Tick which of these are true?)

❗ Check all that apply
Please choose **all** that apply:

- ☐ I don’t have time
- ☐ I don’t have any way to get there (e.g no car/bus)
- ☐ The weather (cold, hot, wet weather)
- ☐ I have a disability or injury
- ☐ My family or I can’t afford it
- ☐ I feel embarrassed about exercising in public
- ☐ I am not good at sports/don’t know how to exercise
- ☐ I don’t have anyone to exercise with
- ☐ I don’t enjoy exercise/sports
- ☐ My mood/mental health
- ☐ I don’t have anywhere safe to go
- ☐ My religion/culture
- ☐ Nothing
- ☐ Other: ________

What motivates you to be physically active and/or play sports?

Only answer this question if the following conditions are met:
Answer was 'No' at question '1 [surveyFilter]' (Are you currently under a child protection/guardianship order (living away from your parent/s)?) *and* Answer was 'No one will know I’ve done this survey' at question '2 [filter1]' (Tick which of these are true?) *and* Answer was 'If I start the survey I can stop at anytime and no one will know' at question '3 [filter2]' (Tick which of these are true?)

❗ Check all that apply
Please choose **all** that apply:

- ☐ My family encourage me
- ☐ My friends encourage me
- ☐ I have safe places to exercise in bad weather
- ☐ I can get vouchers to help me play organised sport e.g. “Ticket to Play”
- ☐ To be physically healthy
- ☐ To be mentally healthy
- ☐ To improve my body image
- ☐ To meet people
- ☐ I enjoy being physically active/playing sport
- ☐ Being good at sport is important to me
- ☐ To improve my sleep
- ☐ Nothing
- ☐ Other: ____________

WHO do you want to get information from about physical activity and healthy eating?

Only answer this question if the following conditions are met:
Answer was 'No' at question '1 [surveyFilter]' (Are you currently under a child protection/guardianship order (living away from your parent/s)?) *and* Answer was 'No one will know I've done this survey' at question '2 [filter1]' (Tick which of these are true?) *and* Answer was 'If I start the survey I can stop at anytime and no one will know' at question '3 [filter2]' (Tick which of these are true?)

❗ Check all that apply
Please choose **all** that apply:

- ☐ Psychologist/Counsellor at headspace
- ☐ Health professional (GP, nurse)
- ☐ Dietitian, exercise professional e.g physio
- ☐ School
- ☐ Work
- ☐ Parents
- ☐ Friends
- ☐ Family (e.g brother, sisters, aunt etc)
- ☐ Other: ______________

HOW do you want get information about physical activity and healthy eating?

Only answer this question if the following conditions are met:
Answer was 'No' at question '1 [surveyFilter]' (Are you currently under a child protection/guardianship order (living away from your parent/s)?) *and* Answer was 'No one will know I've done this survey' at question '2 [filter1]' (Tick which of these are true?) *and* Answer was 'If I start the survey I can stop at anytime and no one will know' at question '3 [filter2]' (Tick which of these are true?)

❗ Check all that apply
Please choose **all** that apply:

- ☐ Socials e.g facebook
- ☐ Instagram/Youtube e.g influencers/celebrities
- ☐ Apps
- ☐ Websites
- ☐ Email
- ☐ Talking (face to face/phone/online ie zoom skype)
- ☐ In person groups/programs e.g at PCYC
- ☐ TV
- ☐ Text messages
- ☐ Poster/flyers
- ☐ Books/magazines
- ☐ Other: ______

Questions About You

Now we'd like to ask some questions about you, don't worry this is anonymous

What is your age?

Only answer this question if the following conditions are met:
Answer was 'No' at question '1 [surveyFilter]' (Are you currently under a child protection/guardianship order (living away from your parent/s)?) *and* Answer was 'No one will know I've done this survey' at question '2 [filter1]' (Tick which of these are true?) *and* Answer was 'If I start the survey I can stop at anytime and no one will know' at question '3 [filter2]' (Tick which of these are true?)

❗ Only numbers may be entered in this field.
Please write your answer here:

______

What is your gender?

Only answer this question if the following conditions are met:
Answer was 'No' at question '1 [surveyFilter]' (Are you currently under a child protection/guardianship order (living away from your parent/s)?) *and* Answer was 'No one will know I've done this survey' at question '2 [filter1]' (Tick which of these are true?) *and* Answer was 'If I start the survey I can stop at anytime and no one will know' at question '3 [filter2]' (Tick which of these are true?)

❗ Choose one of the following answers
Please choose **only one** of the following:

- ◯ Male
- ◯ Female
- ◯ Other

What is your Living situation?

Only answer this question if the following conditions are met:
Answer was 'No' at question '1 [surveyFilter]' (Are you currently under a child protection/guardianship order (living away from your parent/s)?) *and* Answer was 'No one will know I've done this survey' at question '2 [filter1]' (Tick which of these are true?) *and* Answer was 'If I start the survey I can stop at anytime and no one will know' at question '3 [filter2]' (Tick which of these are true?)

❗ Choose one of the following answers
Please choose **only one** of the following:

- ◯ Living at home with both parents/step-parents
- ◯ living at home with 1 parent
- ◯ Living out of home by myself or with others
- ◯ I'm in short term or unstable accommodation
- ◯ I'm homeless/sleeping rough

What suburb or town do you live in?

Only answer this question if the following conditions are met:
Answer was 'No' at question '1 [surveyFilter]' (Are you currently under a child protection/guardianship order (living away from your parent/s)?) *and* Answer was 'No one will know I've done this survey' at question '2 [filter1]' (Tick which of these are true?) *and* Answer was 'If I start the survey I can stop at anytime and no one will know' at question '3 [filter2]' (Tick which of these are true?)

Please write your answer here:

[ ]

Tick how many apply to you.

Only answer this question if the following conditions are met:
Answer was 'No' at question '1 [surveyFilter]' (Are you currently under a child protection/guardianship order (living away from your parent/s)?) *and* Answer was 'No one will know I've done this survey' at question '2 [filter1]' (Tick which of these are true?) *and* Answer was 'If I start the survey I can stop at anytime and no one will know' at question '3 [filter2]' (Tick which of these are true?)

❶ Check all that apply
Please choose **all** that apply:

- ☐ Go to School/TAFE/University/other education
- ☐ Go to work full time
- ☐ Go to work part time
- ☐ Don't go to school/TAFE/University or work
- ☐ Get Centrelink Payments
- ☐ Home/parenting duties
- ☐ Other: ____________

I am Aboriginal and/or Torres Strait Islander

Only answer this question if the following conditions are met:
Answer was 'No' at question '1 [surveyFilter]' (Are you currently under a child protection/guardianship order (living away from your parent/s)?) *and* Answer was 'No one will know I've done this survey' at question '2 [filter1]' (Tick which of these are true?) *and* Answer was 'If I start the survey I can stop at anytime and no one will know' at question '3 [filter2]' (Tick which of these are true?)

Please choose **only one** of the following:

- ○ Yes
- ○ No

How many times have you been to or spoken to headspace in the past 12 months?

Only answer this question if the following conditions are met:
Answer was 'No' at question '1 [surveyFilter]' (Are you currently under a child protection/guardianship order (living away from your parent/s)?) *and* Answer was 'No one will know I've done this survey' at question '2 [filter1]' (Tick which of these are true?) *and* Answer was 'If I start the survey I can stop at anytime and no one will know' at question '3 [filter2]' (Tick which of these are true?)

❶ Only numbers may be entered in this field.
Please write your answer here:

____

Have you been given a mental health diagnosis by a doctor, psychologist or counsellor?

Only answer this question if the following conditions are met:
Answer was 'No' at question '1 [surveyFilter]' (Are you currently under a child protection/guardianship order (living away from your parent/s)?) *and* Answer was 'No one will know I've done this survey' at question '2 [filter1]' (Tick which of these are true?) *and* Answer was 'If I start the survey I can stop at anytime and no one will know' at question '3 [filter2]' (Tick which of these are true?)

Please choose **only one** of the following:

- ◯ Yes
- ◯ No

If you answered 'Yes' to above question, what is your diagnosis?

Only answer this question if the following conditions are met:
Answer was 'Yes' at question '33 [q27]' (Have you been given a mental health diagnosis by a doctor, psychologist or counsellor?) *and* Answer was 'No' at question '1 [surveyFilter]' (Are you currently under a child protection/guardianship order (living away from your parent/s)?) *and* Answer was 'No one will know I've done this survey' at question '2 [filter1]' (Tick which of these are true?) *and* Answer was 'If I start the survey I can stop at anytime and no one will know' at question '3 [filter2]' (Tick which of these are true?)

❗ Check all that apply
Please choose **all** that apply:

- ☐ Depression
- ☐ Anxiety
- ☐ Other: ______________________

Once you press submit you cannot withdraw from the study.

After you submit, you can get your free drink voucher and can leave your details for the prize draw.

Only answer this question if the following conditions are met:
Answer was 'No' at question '1 [surveyFilter]' (Are you currently under a child protection/guardianship order (living away from your parent/s)?) *and* Answer was 'No one will know I've done this survey' at question '2 [filter1]' (Tick which of these are true?) *and* Answer was 'If I start the survey I can stop at anytime and no one will know' at question '3 [filter2]' (Tick which of these are true?)

{if(surveyFilter.NAOK == "Y", 'If you would like to participate in this study will need a guardian to sign a consent form for you to do the study. Please contact heather on 6324 4048 or heather.bridgman@utas.edu.au (mailto:heather.bridgman@utas.edu.au)', if(filter1.NAOK == 1 or filter2.NAOK == 1,'

Please read the information below and try again.

5. What will I be asked to do

You will be asked to fill in an online survey. The survey will ask questions about the food you eat, what you think healthy food is, where and how you can get healthy food information, what you do for physical activity, what stops you and helps you being physically active and how you want to get information. We will also ask you how many times you've been to headspace, if you have a mental health diagnosis, a few questions about your living situation and if you're working or studying. This survey will take you about 15-20 mins to complete. All information collected will be anonymous. ***This means that no one will know that you have participated.*** headspace won't know if you've participated or not.

8. What if I change my mind during or after the study?

You can stop the survey at any time but once you press 'submit' you can't withdraw that information.

https://surveys2.utas.edu.au/index.php/884437?lang=en (https://surveys2.utas.edu.au/index.php/884437?lang=en)

',if(filter1.NAOK == 3 or filter2.NAOK == 3,'Please read the information sheet or contact 6324 4048 for help', '

Thank you for your time.

Remember no-one will know that you have done this study

Click the link below to leave your details to get your drink voucher, to go into the prize draw for a Fitbit Aspire or $50 gift card and to participate in a one-to-one chat with the researchers.

https://surveys2.utas.edu.au/index.php/515396?lang=en (https://surveys2.utas.edu.au/index.php/515396?lang=en)

')))}
15.06.2021 – 03:35

Submit your survey.
Thank you for completing this survey.
