# Supplementary material for: The knowledge, barriers and opportunities to improve nutrition and physical activity amongst young people attending an Australian youth mental health service: a mixed-methods study
Source: BMC Health Serv Res. 2022 Jun 17;22:789. doi: 10.1186/s12913-022-08182-0 (PMC9205652; doi:10.1186/s12913-022-08182-0)
Supplement: Supplementary file 2 — Additional file 2. [file 12913_2022_8182_MOESM2_ESM.docx]

**Understanding Healthy Eating and Physical Activity Beliefs, Behaviours and Needs Of Young People Accessing Headspace Launceston**

Focus group topic guide

Script

*Hi my name is [name], I’m a clinical and health psychologist.*

*Thank you for taking part in this focus group . Have you all read through the information sheet? Do you have any questions?*

*Today's focus group is confidential so whatever is said in this room needs to stay in this room. I'd ask that you all respect each others privacy .*

*If at any time the questions that I'm asking make you feel stressed or uncomfortable just let me know or you can leave and you don't need to say anything or explain yourself. This focus group is completely voluntary and whether you participate or not will not impact on you your role with headspace or getting support from headspace. If I notice if anyone look uncomfortable I’ll check with to see how you are going. You also don’ t have to answer all the questions if you don’t want to.*

*One more thing, because I’m a psychologist I have a duty of care to report if anyone expresses a risk of harm to themselves or to some else during this focus group.*

*Does anyone have any questions? Is everyone happy to take part?*

*Is everyone ok with being recorded? Ok let’s get started:*

Q1. When we use the words healthy food or good nutrition what does that mean to you ?

Q2. when we use the words physical activity what does that mean to you ?

Q3. How important are physical activity and healthy eating in managing mental health conditions?

Q3. How do YP currently access information about healthy eating and physical activity? (prompt online, apps, peers, family, counsellor)

Q4. How do you think YP want to access information about healthy eating and physical activity? (prompt online, apps, peers, family, counsellor

Q5. what would it be like for YP to talk about healthy foods and physical activity during an appointment at headspace?

Q3. What gets in the way of YP accessing and eating healthy food?s

Q4. What helps YP to accessing and eating healthy foods?

Q5. What gets in the way of YP being physically active? (prompt time, confidence, motivation, cost, not knowing where to go)

Q6. What helps or would help YP participate in or increase physical activity?

Interview topic Guide

*Thank you for taking part in this interview . Have you all read through the information sheet? Do you have any questions?*

*If at any time the questions that I'm asking make you feel stressed or uncomfortable just let me know or you can just stop and you don't need to say anything or explain yourself. This interview is completely voluntary and whether you participate or not will not impact on you getting support from headspace. If I notice you look uncomfortable I’ll check with to see how you are going. You also don’ t have to answer all the questions if you don’t want to.*

*One more thing, because I’m a psychologist I have a duty of care to report if anyone expresses a risk of harm to themselves or to some else during this focus group.*

*Do you have any questions? Are you happy to take part?*

*Are you ok being recorded? Ok let’s get started:*

Q1. What do the words healthy eating mean to you ?

Q2. What do the words physical activity what does that mean to you ?

Q3. How important are physical activity and healthy eating in managing mental health conditions?

Q3. How do you currently access information about healthy eating and physical activity? (prompt online, apps, peers, family, counsellor)

Q4. How would you like to access information about healthy eating and physical activity? (prompt online, apps, peers, family, counsellor

Q5. What would it be like for you to talk about healthy foods and physical activity during an appointment at headspace?

Q3. What gets in the way of you accessing and eating healthy foods?

Q4. What helps you to accessing and eating healthy foods?

Q5. What gets in the way of you being physically active? (prompt time, confidence, motivation, cost, not knowing where to go)

Q6. What helps you or would help you to participate in physical activity?
